# Wireless, Flexible, Ion-Selective Electrode System for Selective and Repeatable Detection of Sodium

**DOI:** 10.3390/s20113297

**Published:** 2020-06-10

**Authors:** Hyo-Ryoung Lim, Yun-Soung Kim, Shinjae Kwon, Musa Mahmood, Young-Tae Kwon, Yongkuk Lee, Soon Min Lee, Woon-Hong Yeo

**Affiliations:** 1 George W. Woodruff School of Mechanical Engineering, Institute for Electronics and Nanotechnology, Georgia Institute of Technology, Atlanta, GA 30332, USA; hlim308@gatech.edu (H.-R.L.); ysk@me.gatech.edu (Y.-S.K.); skwon64@gatech.edu (S.K.); musamahmood@gatech.edu (M.M.); ykwon87@gatech.edu (Y.-T.K.); 2 Department of Biomedical Engineering, Wichita State University, Wichita, KS 67260, USA; yongkuk.lee@wichita.edu; 3 Department of Pediatrics, Gangnam Severance Hospital, Yonsei University College of Medicine, Seoul 06273, Korea; smlee@yuhs.ac; 4 Wallace H. Coulter Department of Biomedical Engineering, Georgia Institute of Technology and Emory University School of Medicine, Atlanta, GA 30322, USA; 5 Parker H. Petit Institute for Bioengineering and Biosciences, Institute for Materials, Neural Engineering Center, Institute for Robotics and Intelligent Machines, Georgia Institute of Technology, Atlanta, GA 30332, USA

**Keywords:** wireless flexible sensor system, ion-selective electrode, sodium detection, carbon–polymer composite transducer

## Abstract

Wireless, flexible, ion-selective electrodes (ISEs) are of great interest in the development of wearable health monitors and clinical systems. Existing film-based electrochemical sensors, however, still have practical limitations due to poor electrical contact and material–interfacial leakage. Here, we introduce a wireless, flexible film-based system with a highly selective, stable, and reliable sodium sensor. A flexible and hydrophobic composite with carbon black and soft elastomer serves as an ion-to-electron transducer offering cost efficiency, design simplicity, and long-term stability. The sensor package demonstrates repeatable analysis of selective sodium detection in saliva with good sensitivity (56.1 mV/decade), stability (0.53 mV/h), and selectivity coefficient of sodium against potassium (−3.0). The film ISEs have an additional membrane coating that provides reinforced stability for the sensor upon mechanical bending. Collectively, the comprehensive study of materials, surface chemistry, and sensor design in this work shows the potential of the wireless flexible sensor system for low-profile wearable applications.

## 1. Introduction

Wireless, flexible film-based ion-selective electrodes (ISEs) have great potential for replacing existing bulky equipment with a miniaturized and mobile system for many clinical applications based on noninvasive measurements [1,2,3]. Selective detection of sodium in sweat and saliva, correlated to its level in blood [4], has been used for disease diagnosis (e.g., cystic fibrosis and cardiovascular risk) [5,6]. However, it is still very challenging for flexible film ISEs to offer repeatable and accurate measurements over an extended period since they are vulnerable to chemical and mechanical deformation. For instance, flexible film ISEs often lose their sensing capabilities after conditioning, which is an essential processing step that ensures sensor stability, owing to leakages from the softening of the surrounding materials [7,8,9]. There are recent studies enabling disposable wireless film systems [10,11], whereas the long-term stability of flexible ISEs requires complicated microfabrication processes and facilities that are highly expensive and time-consuming [12].

Structural deterioration is the root cause that prevents flexible ISEs from repeatable and accurate measurements even during a short period of electrochemical testing. In order to maximize sensor lifetime, many studies focused on the proper wetting between hydrophobic ion-selective membranes (ISMs) and bottom transducers to suppress water-layer formation between the two layers [13,14]. However, poor insulation at the interface with shrouding materials has not been fully considered. Given that most of ISEs use tetrahydrofuran as a solvent, which dissolves a wide range of nonpolar and polar chemical compounds, shrinkage and swelling of the insulating structure upon drying may lead to the decreased lifetime of the film ISEs.

Structural deformation is highly important when a polymer composite transducer is involved. Recent studies have introduced carbon-based materials (i.e., carbon black, carbon nanotube, and graphene) mixed with various polymers. The significance of using such composite mixture is easy handling and processing on the flexible curved platforms (e.g., flexible circuits, wearables, and fabrics); however, to the best of our knowledge, none of the carbon-based composite transducers has been applied to flexible electronics [7,14,15,16,17,18,19,20,21,22,23,24]. In this case, a silicone elastomer (Ecoflex) is promising as a polymer matrix because of its high stretchability and biocompatibility, of which a composite with carbon was extensively studied as a strain sensor in flexible wearables [25,26,27,28,29]. Considering that silicone materials swell significantly in contact with a tetrahydrofuran (THF) solvent [30,31], proper insulation that endures polymeric shrinkage should be achieved.

Here, this paper demonstrates the applicability and reusability of a wireless, flexible, film ISE system for sodium detection in saliva. A sodium sensor is integrated on the microstructured contact pads of a flexible circuit. A working electrode in the sodium sensor is formed by a hydrophobic carbon black (CB)/silicone elastomer composite and highly selective ISM, along with robust chemical insulation. The CB/polymer, in its novel implementation as an ion-to-electron transducer, provides cost efficiency, design flexibility, and shelf life up to several months. The optimal membrane ingredients in the ISM enable the flexible ISE to achieve good selectivity and accuracy in detecting sodium among monovalent ions [32]. Moreover, we show that a subsequent membrane coating to a film reference electrode (RE) compensates a decrease in linear activity from repetitive mechanical bending. As a result, the flexible sensor system enables the highly accurate and selective detection of sodium in saliva (6.5 to 11.8 mM) for over 3 weeks.

## 2. Materials and Methods

### 2.1. Materials

4-tert-Butylcalix [4] arene-tetraacetic acid tetraethyl ester (sodium ionophore X), bis(2-ethylhexyl) sebacate (DOS), poly(vinyl chloride) (PVC), tetrahydrofuran (THF), potassium tetrakis(p-chlorophenyl)borate (KTClPB), hydrochloric acid (HCl), and polyvinyl butyral (PVB) were purchased from Sigma Aldrich. Sodium tetrakis-[3,5-bis(trifluoromethyl)phenyl] borate (NaTFPB) was purchased from Alfa Aesar. Sodium chloride was purchased from Acros Organics, and potassium chloride, calcium chloride dihydrate, and magnesium chloride hexahydrate were from Fisher Chemical. Ecoflex 00-30 was purchased from Smooth-On, and carbon black (CB, Vulcan XC 72R) was obtained from FuelCellStore. Noncyanide silver solution (RioGrande) was used to electroplate a thin Ag film for the solid-state RE.

### 2.2. Flexible-Circuit Design

A pair of electrodes for sodium sensing were integrated onto a flexible circuit. The circuit design followed our prior works [33,34], including a Bluetooth low-energy chip, 2.45 GHz chip antenna, and a rechargeable battery. The flexible circuit was utilized for reading potential differences between electrodes changed in different sodium concentrations and wireless transmission of sensing data (details of the circuit design and list of components in Figure S1 and Table S1).

### 2.3. Electrode Fabrication

The CB/Ecoflex composite was prepared by mixing 9 wt% CB and 91% Ecoflex 00-30 in 15 g of isopropyl alcohol (IPA) by stirring for 30 min at 600 rpm. After complete mixing, the composite paste was simply glued or printed on any substrates during several months of storage at room temperature. Three different substrates were used as electron conductors: Teflon body Au disk (BASi, 3 mm in diameter), Au e-beam evaporated Si wafer, and a Cu pad with a polyimide (PI) shroud on the flexible circuit. The CB/Ecoflex composite transducer was glued on different substrates and cured at 150 °C for 1 h. On the flexible circuit, we electrochemically formed silver/silver chloride (Ag/AgCl) RE on the other Cu pad of the circuit. Galvanostatic deposition of Ag was performed at 10 mA/cm^2^ for 3 min with a stainless-steel CE/RE. After cleaning with DI water, the Ag surface was chlorinated in a 0.1 M KCl and 0.01 M HCl solution at 1 mA/cm^2^ for 1 min.

### 2.4. Membrane-Cocktail Fabrication

The CB/Ecoflex electrodes were coated with sodium ISM after complete drying. Two types of sodium ISM were used: (i) sodium ionophore X (2.67 mg), DOS (174.53 mg), PVC (88 mg), NaTFPB (1.47 mg) in 2 mL of THF; and (ii) sodium ionophore X (2.0 mg), DOS (200 mg), PVC (100 mg), and KTClPB (0.50 mg) in 2 mL of THF. The molar ratio of ionophore/NaTFPB and ionophore/KTClPB was 1.62:1 and 2:1, respectively. The mixtures were vortexed for 1 h to make a homogeneous solution. The 2 µL ISM was dropped on the electrodes. The Ag/AgCl film RE, formed on a Cu pad of the circuit, was coated with a membrane cocktail composed of 78.1 mg PVB, 50 mg sodium chloride, and 1 mL methanol [35]. The resulting ISEs and RE were dried at room temperature overnight.

### 2.5. Measurement of Sensing Capabilities

Open-circuit potential was measured from a two-electrode system that included the different types of working electrodes along with the RE. A commercial solid-state RE (NT MRX11) was used to measure the signal voltage from the Au disk and Au/Si electrodes. Sodium chloride solutions with different concentrations were used to obtain sensitivity information (10^−7^ to 1 M). Considering a normal level in human saliva is 4 to 37 mM, solutions with 10^−4^ to 1 M were used for repeatability and selectivity against potassium, magnesium, and calcium ions in the corresponding chloride cation solutions. For testing long-term repeatability and selectivity, we conducted overnight conditioning before initiating measurements (when the sensor was fully dried) and at least three times cleaning upon repetitive measurements to remove any ion sources from the surface. Comparison of the voltage response to the commercial sensor was performed by using an all-solid-state sodium ISE (CNT_ISE M023) and RE (NT MRX11). Saliva samples were collected during each calibration protocol and stored less than 1 h before analysis. Chronopotentiometry was conducted for 60 s at ± 1 nA. Before measurement, the surfaces were soaked for 30 min for conditioning. All sensor measurements were conducted with a Gamry potentiostat (Interface 1010E, Gamry Instruments Inc., Warminster, PA, USA).

## 3. Results and Discussion

### 3.1. Wireless Flexible Sodium Sensor for Sodium Detection in Saliva

**Figure 1:**
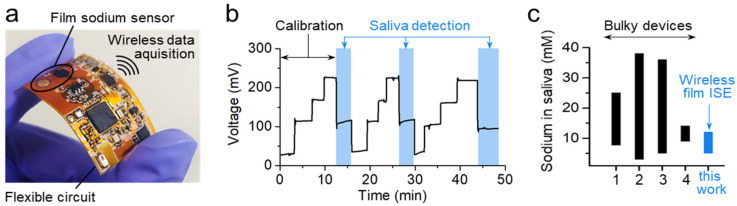
Wireless, flexible sodium ion-selective-electrode (ISE) system. (**a**) Photograph of wireless, flexible electronic system with all-solid-state ISE and reference electrode (RE). (**b**) Repeated measurements of signal voltages in calibration sodium solutions (concentrations: 10^−4^, 10^−2^, 10^−1^, and 1 M) and human saliva. (**c**) Comparison of measured sodium levels between commercial bulky devices (1–4) and our wireless system, where reference 1: 8.7–24 mM (generally known) [36], reference 2: 4–37 mM (photometer) [37], reference 3: 6–35 mM (electrochemical luminescence) [5], reference 4: 10–13 mM (electrochemical ISE) [6], and our wireless film ISE: 6.5–11.8 mM.

Figure 1 shows an overview of a film-type sodium sensor integrated with a flexible wireless electronic system and its sensing capability for saliva. The electronic device included a pair of all-solid-state ISE and RE using Ag/AgCl, an analog-to-digital converter for data acquisition and conversion, and a wireless data-transmission unit (Figure 1a). Overall, the low-profile device with 3 × 2 × 0.2 cm in size and 2 g in weight exhibited great potential to be used as a portable sensor system for various types of wearable applications. While real-time detection was enabled by deploying a Bluetooth low-energy module, a thin selective membrane electrode directly formed on a Cu contact pad on the flexible circuit showed excellent selectivity and long-term stability of sensitivity desired for repeatable use. The flexible sodium sensor exhibited near-Nernstian sensitivity of 56 mV/decade in sodium concentrations from 10^−4^ to 1 M, covering a typical spectrum of saliva [5,6,36,37].

Figure 1b demonstrates the good repeatability of voltage signal from the film electrodes based on calibration protocols and corresponding saliva detection, multiple times. The developed thin-film sensor also showed long-term stable data recording with minimal change in voltage (0.53 mV/h; n = 3) adopting a chemically resistant layer around the electrodes (Figure S2; sodium concentration: 10^−2^ M). As illustrated in Figure 1c, the resulting saliva concentration was in the range of the reported values analyzed by commercial bulky devices. Specifically, normal saliva sodium level is in the range of 4 to 37 mM [36,37] and 6 to 35 mM [5,6] via photonic and electrochemical detection, respectively. The measured sodium concentration from our sensor ranged from 6.5 to 11.8 mM (7 times), which was consistent with the reported range. The film sensor showed good sensitivity and saliva-sensing capability when compared with those for the commercial all-solid-state ISE and RE (56 mV/decade and 3 to 13 mM sodium in saliva; Figure S3). Unlike the case of bulky devices, the thin-film ISE in this work showed great applicability for a wearable, portable system, along with wireless data acquisition, mechanical flexibility, and repeatable sensing capabilities.

### 3.2. Design and Fabrication of Film ISE

**Figure 2:**
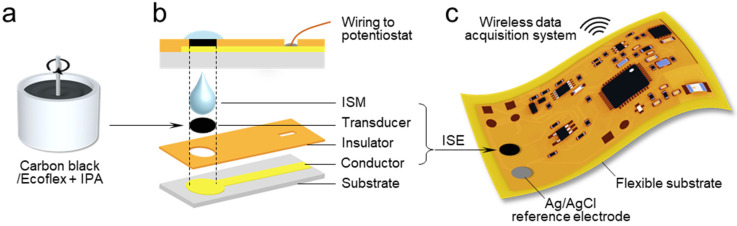
Fabrication process of an all-solid-state film ISE. (**a**) Schematic illustration showing simple procedure for a CB/Ecoflex composite with long-term (several months) storage at room temperature (RT). (**b**) Illustrated structure of film ISE, including deposited ion-selective membrane (ISM), CB/Ecoflex transducer, insulator, and Au conductor on Si wafer. (**c**) Direct integration of fabricated ISE and Ag/AgCl reference electrode on Cu pads in the flexible circuit for wireless data acquisition.

Figure 2 shows fabrication procedures for the film ISE, and verification strategies for the flexible structural designs. First, a highly flexible and biocompatible CB/Ecoflex composite was prepared as an ion-to-electron transducer. As depicted in Figure 2a, the composite transducer was simply mixed with IPA by a mechanical stirrer where all the ingredients were biocompatible to be used in wearable sensors. To compare different insulation effects, Ecoflex 00-30, PVC, and PI were shrouded around the Au/Si electrode. The verified functionality of the composite transducer is shown in Figure S4. When formed on a standard Teflon-shrouded Au disk electrode, the CB/Ecoflex/ISM showed a sensitivity of 57 mV/decade from 10^−5^ to 1 M sodium with a detection limit of 4 × 10^−6^ M. There was a potential drift (ΔE/Δt) of 140 μV/s, measured by the chronopotentiometry technique. The low-frequency capacitance (C_L_) of the CB/Ecoflex solid contact was calculated by ΔE/Δt = I/C_L_ and estimated as 7.14 μF. This value was relatively low compared to that of conducting polymers [38], but higher than that of the polymer/carbon case (polyaniline/graphene, 0.29 μF) [15]. Overall, the sensing performance of the film ISE was compatible with conventional ISEs based on carbon–polymer transducers (Table S2). The prepared carbon composite was laminated on a microfabricated Au conductor (Figure 2b) and on the Cu pad of the flexible circuit (Figure 2c). Then, it was cured at 150 °C for 1 h, followed by the coating of a small volume of sodium ISM on top of the transducer. Importantly, the ISE directly formed on the circuit removed contact instability, and the need for space and carrying capacity to use external equipment. Collectively, the fabrication method of the CB/Ecoflex composite transducer provides cost efficiency, design flexibility, and simplicity in storage and handling.

### 3.3. Characterization of CB/Ecoflex Composite ISE

#### 3.3.1. Effect of Insulating Layer

**Figure 3:**
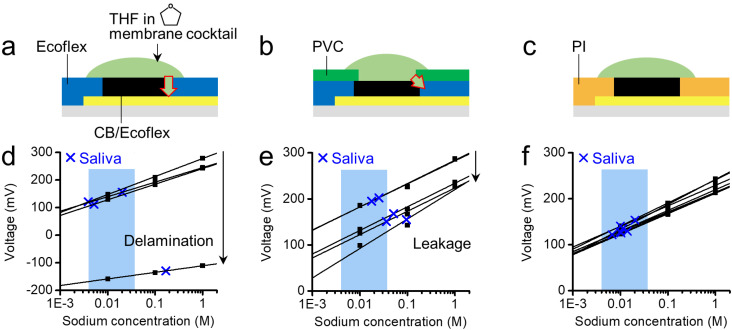
Analysis of repeatability in sodium sensing with different insulating layers. (**a**–**c**) Schematic diagrams showing interfacial chemical interactions between membrane solvent and insulating layers. Insulating materials: (**a**,**d**) Ecoflex, (**b**,**e**) Ecoflex/ poly(vinyl chloride) (PVC), and (**c**,**f**) polyimide (PI) only. (**d**–**f**) Corresponding sensing data per each case in (**a**–**c**) showing results from both calibration solutions and saliva. Highlighted blue boxes in (**d**–**f**): normal range of sodium concentrations in saliva.

The sensing capability of the film ISEs depends not only on the properties of the distinct layers, but also on their interfacial failure. Any chemical damage at the interfacial region is hardly recoverable, affecting the repeatability of sensors. Here, we provide additional insights into the proper choice of insulating materials with respect to chemical resistance to the membrane solvent. Three ISEs, covered by a different insulating layer, were used for testing repeatability. Figure 3 summarizes the sensing results with different insulators and a solvent (THF) that is widely used in PVC-based ISMs. In Figure 3a, the platinum-catalyzed silicone Ecoflex is shown deformed upon the membrane drying at least overnight. The use of THF typically causes the swelling of silicone, such as Ecoflex and polydimethylsiloxane (PDMS) [30,31], which was observed in a CB/Ecoflex-only case, leading to eventual breakdown due to delamination (Figure S5). Consequently, the sensor lost its initial activity after three uses, resulting in out-of-range sodium levels in saliva (blue highlighted box, Figure 3d).

An additional coating of the core polymer PVC on the top of the interface showed no visible delamination, as a membrane was holding compartment in bulky systems (Figure 3b) [23,39]. However, the voltage response varied in accordance with leakage of the analyte solution during repetitive measurements, causing significant errors in the determination of ion concentrations (Figure 3e). This delamination and leakage failure were triggered by the shrinkage of the polymeric chains at the interfacial region between the PVC and the soft silicone upon drying. A comparison of the PVC film, formed on the Ecoflex to a rigid substrate, showed significant shrinkage, pore formation, and delamination. Consequently, it made the pure PVC inappropriate to be a membrane-holding compartment in the flexible film system (Figure S6). The use of a stable polymer PI in Figure 3c solved this problem since PI is a good insulator with high chemical and electrical resistance. Although the surface of the silicone in the composite transducer may have been affected by the THF, the inorganic CB likely inhibited further deformation during complete drying if insulation was ensured. The measured data in Figure 3f clearly demonstrate the desired characteristics, including repeatable testing, continuous reading both in calibration solution (10^−1^ M) and saliva for 30 min, and long-term stability of sensitivity up to three weeks when stored dried (Figure S7). Therefore, results from this work provide experiment insights into the effect of chemical insulation on the repeated use of film ISEs.

#### 3.3.2. Effect of Membrane Ingredient

**Figure 4:**
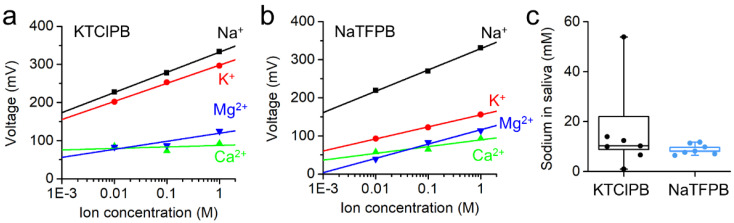
Performance comparison of film ISEs with different anionic sites. Sensitivity and selectivity results based on (**a**) potassium anionic site (KTClPB) and (**b**) sodium anionic site (NaTFPB). (**c**) Comparison of detected sodium levels in saliva measured seven times from both ISEs.

Our system targeted primarily clinical applications where sodium/potassium selectivity played a crucial role in disease diagnosis. To achieve good accuracy, it was essential to choose an appropriate anionic site, as it affected selectivity as well as sensitivity [32,40,41]. We selected two different salts, namely, NaTFPB and KTClPB, widely used in recent works [42,43,44,45,46]. Considering that the extensive conditioning protocol is often disregarded in flexible film electrodes and wearable platforms, we provide the experiment results on using both salts after the same period of conditioning. Figure 4a,b shows the signal voltage plotted against concentrations of sodium, potassium, magnesium, and calcium for different film ISEs that include KTClPB and sodium anionic sites NaTFPB. The selectivity coefficient was calculated by

LogK^pot^_A, B_ = (E_B_ − E_A_)·z_A_F/2.303RT + (1 − z_A_/z_B_)logα_A_, (1)

where E, potential; A, target ion; B, interfering ion; z, valency of ion; F, Faraday’s constant; R, gas constant; T, temperature; and α, activity [47]. Both ISEs showed sodium sensitivity, with the NaTFPB case being capable of the selective detection of sodium (56 mV/decade) against potassium (31 mV/decade) with K^pot^_Na+ K+_ of –3.0, which showed better selectivity than a CNT/PVC-based ISE with NaTFPB (K^pot^_Na+ K+_ = –2.4) [22]. The ISM formed with the KTClPB exhibited relatively bad selectivity (of less than –1), owing to the interfacial dissolution of the potassium ions from the membrane. Furthermore, the film ISEs maintained their ability to detect sodium in saliva upon repeated measurements (55.2 mV/decade; 7 times) and after being stored for 1 week in air (56.1 mV/decade) (Figure S8).

Figure 4c compares the detected sodium levels from both ISEs. While the KTClPB case had a poor performance (1 to 54 mM), the NaTFPB showed good detection of sodium in saliva (6.5–11.8 mM). Table 1 summarizes the sensitivity and selectivity of film ISEs on the basis of different anionic sites. Given that saliva contains proteins/enzymes, as well as electrolytes including ions [48], future work will examine long-term performance when interacting with biomolecules. Furthermore, other components, including the ion ionophore/anionic site ratio, thickness, and conditioning time required for the flexible film platform, will be considered in future work.

### 3.4. Sensor-Performance Characterization with Mechanical Bending

**Figure 5:**
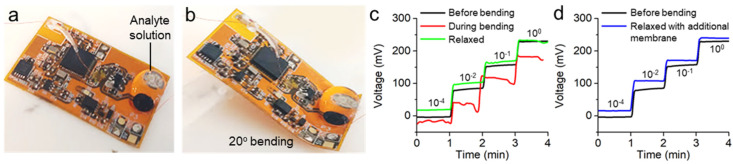
Wireless sodium detection with film ISEs. Photographs of circuit (**a**) before bending and (**b**) during 20° bending. (**c**) Voltage responses with different sodium concentrations for three cases: before bending (black line), during bending (red line), and after bending (green line). (**d**) Comparison of voltage responses, showing the role of additional polyvinyl butyral (PVB) membrane on RE.

In this work, we demonstrated sensor performance with the mechanical bending of the system. The sensor, including the film ISE and Ag/AgCl RE, was directly formed onto the Cu pads of the circuit. As summarized in Figure 5, the film sensor’s performance was measured before bending, under 20° bending, and after relaxation. The measured voltage signals validated a linear response to sodium ion; however, sensor sensitivity (initially 58 mV/decade) showed a substantial decrease under and after bending (48 and 49 mV/decade, respectively). As shown in Figure 5b, the mechanical bending caused degradation of the membrane structure in the sensor, which deteriorated sensor performance (Figure 5c). We observed that the RE membrane had significant deformation. Thus, the follow-up study attempted the reinforcement of two additional PVB–NaCl layers on the deformed RE at relaxed state. Figure 5d captures the effect of the additional membrane coating by showing similar sensing performance compared to the case before bending. The near-Nernstian potential from the reconstructed electrodes confirmed our hypothesis of membrane deformation, supported by strain dependence of key electrochemical parameters [49]. Additionally, given that the small form factor of the circuit leading to the distance between the ISE and RE was very narrow (3 mm), future work will focus on the use of appropriate salt bridges on top of the RE, which hinders the increase of sodium ions in the vicinity of the RE. Table 2 summarizes the comparison of device materials, structures, and sensor performance of this work with prior reports. Overall, the newly developed wireless and flexible ISE showed advantages in portability, sensitivity, and selectivity.

## 4. Conclusions

This paper introduced a wireless, flexible ISE system for highly sensitive, selective, and stable detection of sodium in saliva. A hybrid composite nanostructure, made of CB and soft elastomer, showed stable operation as an ion-to-electron transducer with long-term stability up to several months when stored at room temperature. The wireless, low-profile sensor package showed good sensitivity (56.1 mV/decade), stability (0.53 mV/h), and selectivity coefficient of sodium against potassium (**K^pot^_Na+ K+_**: –3.0). The additional coating of the PVB membrane on the RE enhanced the stability of the wireless sensor during mechanical bending. Collectively, the presented results in this work capture the potential of the miniaturized, wireless flexible sensor system for applications in portable wearable health monitors and clinical studies.

## Figures and Tables

**Table 1:** Sensitivity and selectivity data of film ISEs based on different anionic sites.

Interfering Ion, J	KTClPB		NaTFPB	
	Sensitivity (mV/decade)	K^pot^_Na+ J_	Sensitivity (mV/decade)	K^pot^_Na+ J_
K^+^	47.5	−0.6	31.6	−3.0
Mg^2+^	21.1	−6.6	37.5	−6.9
Ca^2+^	3.9	−7.7	17.7	−7.6
Na^+^	52.9	0	56.1	0

**Table 2:** Comparison of device materials, structure, and performance.

Reference	Substrate	Flexible/Wireless	Sensitivity (mV/decade) ^(1)^	Selectivity (K^pot^_Na+ K+_)	Lifetime
This work	PI	Yes/yes	56.1	–3.0	3 weeks
[50]	PET	Yes/yes	0.031 nF/mM ^(2)^	Not available	Not available
[51]	PDMS	Yes/no	58	Not available	<1 h
[52]	Tape	Yes/no	56.2	Not available	Not available
[53]	Paper	Yes/no	55.7	Not available	Not available
[54]	Fiber	Yes/no	55.1	Not available	4 weeks
[55]	Plastic	Some/no	55.9	−2.2	Not available
[12]	Wafer	Some/no	56.6	−2.5	2 months

^(1)^ All covers sodium level in saliva. ^(2)^ Capacitor-based sensor. PI: polyimide, PET: poly(ethylene terephthalate), and PDMS: polydimethylsiloxane.

## Supplementary Materials

The following are available online at https://www.mdpi.com/1424-8220/20/11/3297/s1. Figure S1: illustration of wireless ion-selective electrode circuit, Figure S2: voltage stability, Figure S3: saliva concentration measured from a commercial solid-state sensor, Figure S4: hydrophobicity and sensor capability of CB/Ecoflex transducer, Figure S5: drying shrinkage, Figure S6: PVC delamination from Ecoflex, Figure S7: long-term stability, Figure S8: repeatable measurement of sodium in saliva, Table S1: list of components used for circuit, and Table S2: performance comparison of carbon–polymer composite-based ISEs.
