# Supplementary material for: Wireless, Flexible, Ion-Selective Electrode System for Selective and Repeatable Detection of Sodium
Source: Sensors (Basel). 2020 Jun 10;20(11):3297. doi: 10.3390/s20113297 (PMC7309126; doi:10.3390/s20113297)
Supplement: Supplementary file 1 [file sensors-20-03297-s001.pdf]

Supplementary Materials

Wireless, Flexible, Ion-Selective Electrode System for Selective and Repeatable Detection of Sodium

Hyo-Ryoung Lim [1], Yun-Soung Kim [1], Shinjae Kwon [1], Musa Mahmood [1], Young-Tae Kwon [1], Yongkuk Lee [2], Soon Min Lee [3], and Woon-Hong Yeo [1,4,5,*]

[1] George W. Woodruff School of Mechanical Engineering, Institute for Electronics and Nanotechnology, Georgia Institute of Technology, Atlanta, GA, 30332, USA; hlim308@gatech.edu (H-.R. L.); ysk@me.gatech.edu (Y-.S.K.); skwon64@gatech.edu (S.K.); musamahmood@gatech.edu (M.M.); ykwon87@gatech.edu (Y-.T.K.)

[2] Department of Biomedical Engineering, Wichita State University, Wichita, KS 67260, USA; yongkuk.lee@wichita.edu

[3] Department of Pediatrics, Gangnam Severance Hospital, Yonsei University College of Medicine, Seoul, South Korea; smlee@yuhs.ac

[4] Wallace H. Coulter Department of Biomedical Engineering, Georgia Institute of Technology & Emory University School of Medicine, Atlanta, GA, 30322, USA

[5] Parker H. Petit Institute for Bioengineering and Biosciences, Institute for Materials, Neural Engineering Center, Institute for Robotics and Intelligent Machines, Georgia Institute of Technology, Atlanta, GA, 30332, USA

* Correspondence: whyeo@gatech.edu (W-.H.Y.); Tel.: +1-404-385-5710; Fax: +1-404-894-1658

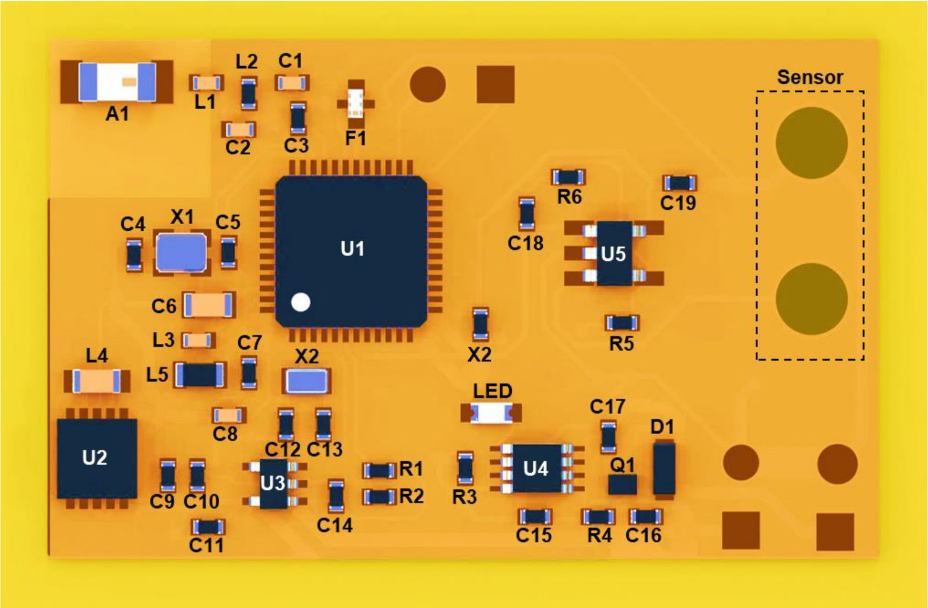

Figure S1. Illustration of wireless ion-selective sodium sensor circuit components with detail list of the surface mount chip components in Table S1. The sensor includes an ion-selective electrode (ISE) and an Ag/AgCl reference electrode (RE), which are directly formed onto Cu-pads of the circuit.

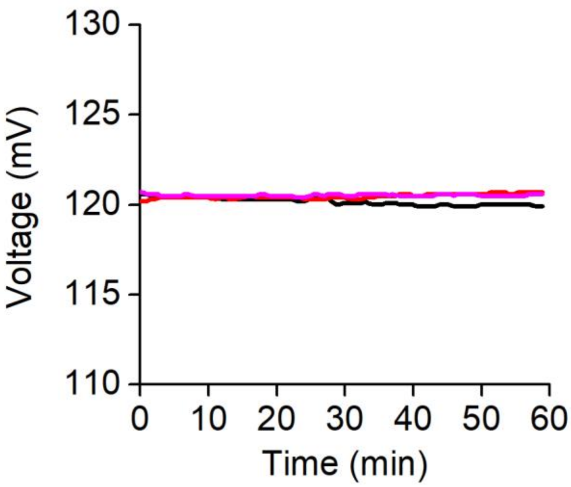

Figure S2. Voltage stability (0.53 mV/h; n = 3) of the all-solid-state film sodium ISE in 10^{-2} M sodium chloride.

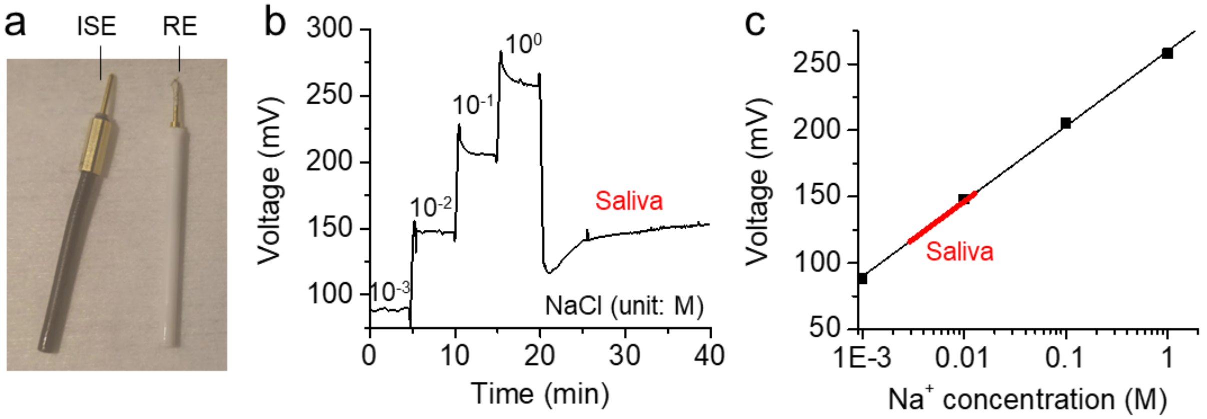

Figure S3. Saliva concentration measured with using a commercial all-solid-state ISE and RE. (a) Photograph of the commercial sodium ISE and RE. (b) Voltage response of the sensor in NaCl solutions and Saliva. (c) Saliva concentration shows 3-13 mM Na^+ (highlighted in red on the calibration line with a slope of 56 mV/decade).

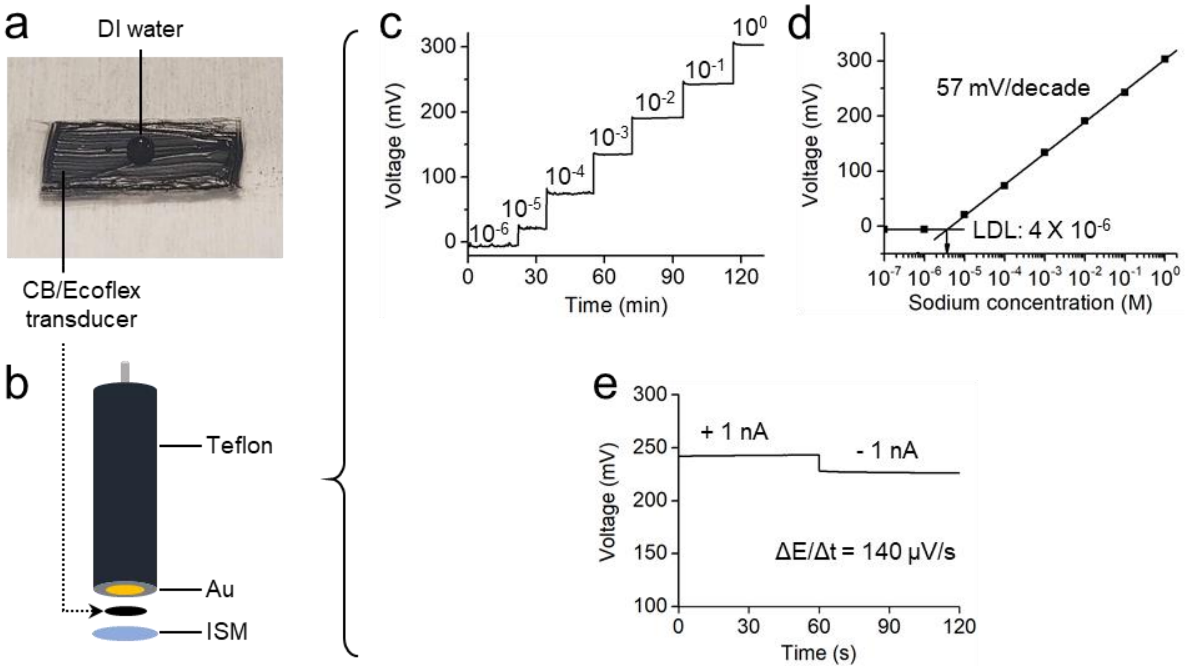

Figure S4. Sensing capabilities of a CB/Ecoflex-based ISE formed on a standard Teflon-body electroe. (a) Photograph showing a hydrophobicity of the CB/Ecoflex composite, ensuring a good contact with the PVC ISM. (b) Schematic diagram of the structure of CB/Ecoflex/ISM formed on an Au disc shrouded by Teflon. Sensing capabilities measured with using the ISE (b): (c) Real-time voltage response in 10^{-6} to 10^{0} sodium chloride solutions, (d) calculated sensitivity and low detection limit (LDL), and (e) voltage stability measured by chronopotentiometry in 10^{-2} sodium chloride.

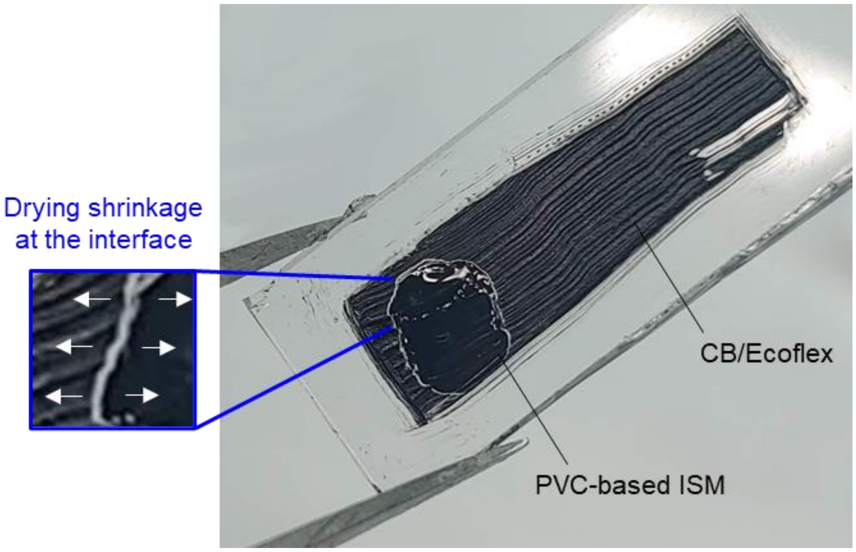

Figure S5. Photograph showing a drying shrinkage at the interface between a CB/Ecoflex transducer and ISM that contains THF, resulted in a delamination.

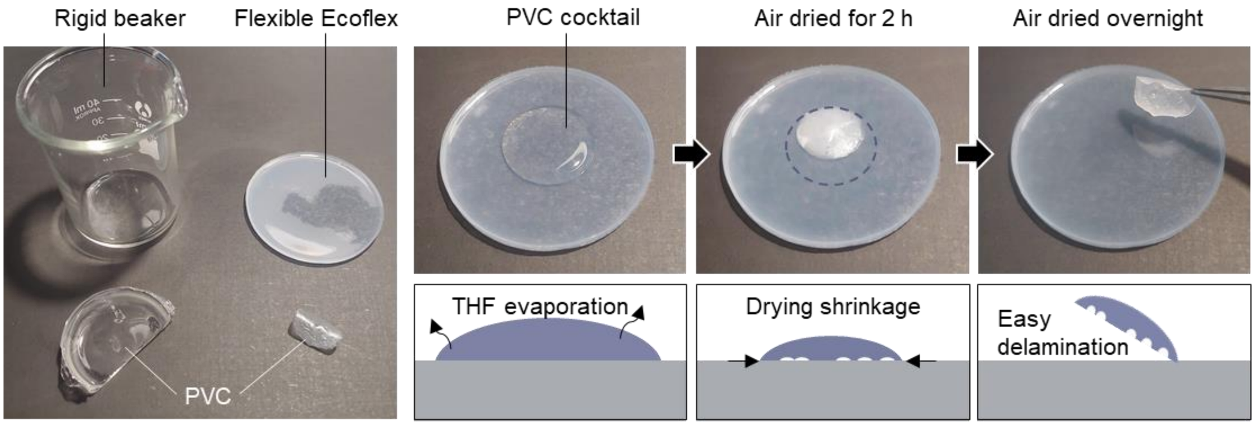

Figure S6. Photographs showing a drying shrinkage of PVC formed on the surface of Ecoflex compared to a rigid beaker. While the PVC cocktail in the beaker was dried stuck at the surface of the rigid glass, the film formed on the flexible substrate shows a significant shrinkage and pores.

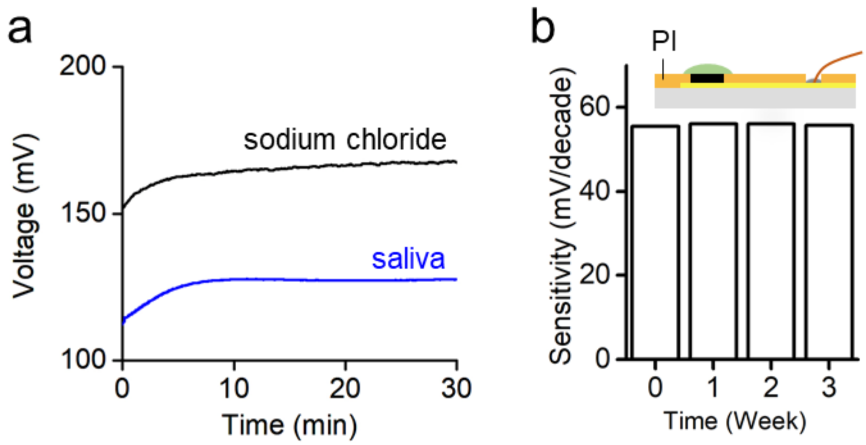

Figure S7. Short-term and long-term stable voltage reading of an all-solid-state film ISE enabled by efficient chemical insulation. (a) Voltage reading of the sensor in 10^{-1} sodium chloride solution and saliva. (b) Long-term stability of stability for three weeks.

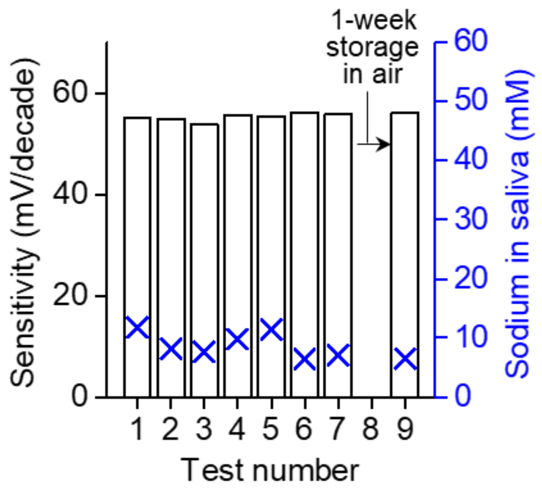

Figure S8. Sensitivity and applicability of sodium detection of our film ISE during 1-week storage in air. The 7 times measurement was conducted continuously.

Table S1. List of surface mount components used in wireless ion-selective sodium sensor circuit.

**Component**	**Description**	**Value**	**Part number**
U1	Bluetooth PSoC	N/A	NRF52832-QFAA-R
U2	3.3 voltage regulator	N/A	TPS63001
U3	Current limit active-low load switch	N/A	TPS22941
U4	Battery recharge	N/A	MCP73831
U5	Single channel Op-amp IC	N/A	AD8603AUJZREEL7
F1	2.45 GHz low pass filter	N/A	2450FM07A0029
A1	2.45 GHz RF chip antenna	N/A	2450AT18A100
D1	Schottky Diode	N/A	641-1285-1-ND
Q1	P-MOSFET	N/A	DMP21D5UFB4-7BDICT-ND
X1	32 MHz crystal	N/A	ECS-320-8-37CKM
X2	32.768 kHz crystal	N/A	ECS-327-9-12-TR
L1	0402 inductor	3.9 nH	N/A
L2	0402 inductor	2.7 nH	N/A
L3	0402 inductor	15 nH	N/A
L4	0603 inductor	2.2 μH	N/A
L5	0603 inductor	10 μH	N/A
C1	0402 ceramic capacitor	1.0 pF	N/A
C2, C8	0402 ceramic capacitor	100 nF	N/A
C3	0402 ceramic capacitor	100 pF	N/A
C4, C5, C12, C13	0402 ceramic capacitor	12 pF	N/A
C6	0603 ceramic capacitor	1.0 μF	N/A
C7, C15, C17	0402 ceramic capacitor	4.7 μF	N/A
C9	0402 ceramic capacitor	22 μF	N/A
C10, C11, C14, C18	0402 ceramic capacitor	10 μF	N/A
C16	0402 ceramic capacitor	1.0 μF	N/A
C19	0402 ceramic capacitor	10 nF	N/A
R1, R2	0402 resistor	1 MΩ	N/A
R3	0402 resistor	2 kΩ	N/A
R4	0402 resistor	100 kΩ	N/A
R5, R6	0402 resistor	10 kΩ	N/A
Battery	Lithium-ion polymer		

Table S2. Comparison of sensing capabilities based on carbon-polymer composite transducers.

**Year[Reference]**	**Transducer**	**Sensitivity mV/decade)**	**Range (M)**	**Ion**
**This work[2020]**	Carbon black/Ecoflex	57.0	10^{-6} to 1	Na^+
**[1][2009]**	CNT/block polymer	58.6	10^{-7} to 10^{-1}	Na^+
**[2][2010]**	CNT/block polymer/ISM	58.2	10^{-6} to 10^{-1}	Na^+
**[3][2019]**	CNT/copolymer	56.3	10^{-7} to 10^{-1}	K^+
**[4][2017]**	CNT/porphyrinoids	60.5	10^{-7} to 10^{-1}	K^+
**[5][2015]**	Graphene/AgTFPB	56.5	10^{-5} to 10^{-2}	K^+

References

1. Zhu, J.; Qin, Y.; Zhang, Y., Preparation of all solid-state potentiometric ion sensors with polymer-CNT composites. *Electrochemistry Communications* **2009,** 11, (8), 1684-1687.
2. Zhu, J.; Li, X.; Qin, Y.; Zhang, Y., Single-piece solid-contact ion-selective electrodes with polymer–carbon nanotube composites. *Sensors and Actuators B: Chemical* **2010,** 148, (1), 166-172.
3. Kałuża, D.; Jaworska, E.; Mazur, M.; Maksymiuk, K.; Michalska, A., Multiwalled Carbon Nanotubes–Poly(3-octylthiophene-2,5-diyl) Nanocomposite Transducer for Ion-Selective Electrodes: Raman Spectroscopy Insight into the Transducer/Membrane Interface. *Analytical Chemistry* **2019,** 91, (14), 9010-9017.
4. Jaworska, E.; Naitana, M. L.; Stelmach, E.; Pomarico, G.; Wojciechowski, M.; Bulska, E.; Maksymiuk, K.; Paolesse, R.; Michalska, A., Introducing Cobalt(II) Porphyrin/Cobalt(III) Corrole Containing Transducers for Improved Potential Reproducibility and Performance of All-Solid-State Ion-Selective Electrodes. *Analytical Chemistry* **2017,** 89, (13), 7107-7114.
5. Sun, Q.; Li, W.; Su, B., Highly hydrophobic solid contact based on graphene-hybrid nanocomposites for all solid state potentiometric sensors with well-formulated phase boundary potentials. *Journal of Electroanalytical Chemistry* **2015,** 740, 21-27.
